# Endovascular treatment of peripheral arterial disease: Endo-STAR framework for the design, conduct, and reporting of trials

**DOI:** 10.1093/bjs/znaf020

**Published:** 2025-04-17

**Authors:** Ewa M Zywicka, Andrew J Moore, Christopher Twine, Christian-Alexander Behrendt, Michel Bosiers, Marianne Brodmann, Edward Choke, Gert J de Borst, Athanasios Diamantopoulos, Florian Enzmann, Alik Farber, Gary Ansel, Dario Gattuso, Gerard S Goh, Goueffic Yann, Shirley Jansen, Mario Landini, Anne Lejay, Michael Lichtenberg, Matthew Menard, Peter Mezes, Joseph Mills, Jane Nixon, Joakim Nordanstig, Kelly O’Connell, Baris Ozdemir, Lorenzo Patrone, Sapna Puppala, Athanasios Saratzis, Eric A Secemsky, Sigrid Nikol, Konstantinos Stavroulakis, Sabine Steiner, Martin Teraa, Isabelle Van Herzeele, Maarit Venermo, Thomas Zeller, Ronelle Mouton, Robert J Hinchliffe

**Affiliations:** Translational Health Sciences, Bristol Medical School, University of Bristol, Bristol, UK; Department of Vascular Surgery, North Bristol NHS Trust, Bristol, UK; Musculoskeletal Research Unit, Bristol Medical School, University of Bristol, Bristol, UK; Translational Health Sciences, Bristol Medical School, University of Bristol, Bristol, UK; Department of Vascular Surgery, North Bristol NHS Trust, Bristol, UK; Department of Vascular and Endovascular Surgery, Asklepios Clinic Wandsbeck, Asklepios Medical School, Hamburg, Germany; Department of Vascular Surgery, University Hospital Bern, University of Bern, Bern, Switzerland; Division of Angiology, Medical University Graz, Graz, Austria; Vascular and Endovascular Surgery Service, Department of General Surgery, Sengkang General Hospital, Singapore; Department of Vascular Surgery G04.129, University Medical Centre Utrecht, Utrecht, The Netherlands; Department of Interventional Radiology, Guy’s and St Thomas’ Hospitals, NHS Foundation Trust, London, UK; Department of Vascular Surgery, Medical University of Innsbruck, Innsbruck, Austria; Division of Vascular and Endovascular Surgery, Boston Medical Center, Boston University Chobanian & Avedisian School of Medicine, Boston, Massachusetts, USA; Healthcare Insights, Columbus, Ohio, USA; Concept Medical Inc., Tampa, Florida, USA; Department of Radiology, Alfred Hospital, Melbourne, Victoria, Australia; Service de chirurgie vasculaire et endovasculaire, Groupe Hospitalier Paris St Joseph, Paris, France; Curtin Medical School, Curtin University, Perth, Western Australia, Australia; Heart and Vascular Research Institute, Harry Perkins Institute of Medical Research, University of Western Australia, Perth, Western Australia, Australia; Clinical and Business Development Europe, Middle East & Africa, Cordis, Miami Lakes, Florida, USA; Department of Vascular Surgery, Kidney Transplantation and Innovation, Strasbourg University Hospital, Strasbourg, France; Vascular Centre, Klinikum Arnsberg, Arnsberg, Germany; Division of Vascular and Endovascular Surgery, Brigham and Women’s Hospital, Harvard Medical School, Boston, Massachusetts, USA; Department of Interventional Radiology, North Bristol NHS Trust, Bristol, UK; Michael E. DeBakey Department of Surgery, Baylor College of Medicine, Houston, Texas, USA; Leeds Institute of Health Sciences, University of Leeds, Leeds, UK; Department of Vascular Surgery, Sahlgrenska University Hospital, Gothenburg, Sweden; Clinical Development Clinical & Medical Affairs, Philips Image Guided Therapy Corporation, Colorado Springs, Colorado, USA; Department of Vascular Surgery, North Bristol NHS Trust, Bristol, UK; Department of Interventional Radiology, West London Vascular and Interventional Centre, Northwick Park Hospital, Harrow, UK; Department of Interventional Vascular Radiology, Leeds Teaching Hospitals NHS Trust, Leeds General Infirmary, Leeds, UK; Department of Cardiovascular Sciences and NIHR Leicester Biomedical Research Centre, University of Leicester, Glenfield General Hospital, Leicester, UK; Division of Cardiology, Beth Israel Deaconess Medical Centre, Boston, Massachusetts, USA; Department of Clinical and Interventional Angiology, Asklepios Klinik St Georg, Hamburg, Germany; Department of Vascular Surgery, University Hospital, LMU Munich, Munich, Germany; Department of Angiology, University Hospital Leipzig, Leipzig, Germany; Department of Vascular Surgery, University Medical Centre Utrecht, Utrecht, The Netherlands; Department of Thoracic and Vascular Surgery, Ghent University Hospital, Ghent, Belgium; Department of Vascular Surgery, University of Helsinki and Helsinki University Hospital, Helsinki, Finland; Abteilung Angiologie, Universitäts-Herzzentrum Freiburg-Bad Krozingen, Bad Krozingen, Germany; Translational Health Sciences, Bristol Medical School, University of Bristol, Bristol, UK; Department of Anaesthesia, North Bristol NHS Trust, Bristol, UK; Translational Health Sciences, Bristol Medical School, University of Bristol, Bristol, UK; Department of Vascular Surgery, North Bristol NHS Trust, Bristol, UK

## Abstract

**Background:**

Endovascular technologies continue to evolve to meet the large and growing burden of peripheral arterial disease. The overall quality of published RCTs in endovascular treatments for peripheral arterial disease is low, resulting in uncertainty over treatment effectiveness. The aim of this study was to develop a framework to improve the design, conduct, and reporting of future clinical trials for infrainguinal endovascular treatments of peripheral arterial disease.

**Methods:**

The authors undertook the design, development, and pilot testing of a novel framework. The study comprised four distinct phases. Phase 1 represented the development of a preliminary framework using content analysis of endovascular interventions described in previously published RCTs. Phase 2 consisted of focus groups with key stakeholders to further develop, revise, and achieve initial consensus on the framework. Phase 3 corresponded to the creation of a modified Delphi questionnaire to achieve final consensus on the framework. Phase 4 included cognitive interviews with professionals designing or undertaking endovascular lower limb trials to pilot test the framework.

**Results:**

Content analysis of 228 endovascular interventions from 112 RCTs identified six key themes, relevant to endovascular peripheral arterial disease interventions, for the framework: expertise; setting; anaesthesia; imaging; intervention components (access; crossing lesion; treating lesion (lesion preparation; intervention; intervention optimization; bailout intervention; and treatment of non-target lesions); and closure of artery); and pharmacological interventions. Further refinements were made to the framework as a result of feedback from three focus groups and a Delphi questionnaire. The framework deconstructs an endovascular intervention into its component parts. The final framework can be accessed at www.endo-star.com. Pilot testing evaluated comprehension, clarity, and completeness of interpretation.

**Conclusion:**

The Endo-STAR framework deconstructs endovascular interventions into their key component parts and has been designed and pilot tested to enhance the quality of RCTs of endovascular interventions in peripheral arterial disease. It may be used to assist in developing future trial protocols, the standardization of infrainguinal endovascular interventions, the monitoring of adherence to the trial protocol, and as a standardized reporting guideline.

## Teaser

The Endo-STAR framework breaks down endovascular interventions into their key component parts. It may be used to assist in developing future trial protocols, the standardization of infrainguinal endovascular interventions, the monitoring of adherence to the trial protocols, and as a standardized reporting guideline.

## Introduction

The Global Burden of Disease Study 2019 estimated that 113 million people over the age of 40 years live with peripheral arterial disease (PAD), a prevalence of 1.52%^1^. There has been a 72% increase in prevalence compared with 1990 and a significant increase in the global prevalence of diabetes mellitus combined with ageing of the Western population is expected to cause a further parallel increase in the prevalence of PAD^2^. Consequently, the need for lower extremity revascularization procedures is also expected to increase^3,4^.

Endovascular interventions are evolving and are now the most common revascularization techniques for lower extremity PAD to improve health-related quality of life and prevent limb loss^5^. However, uncertainties exist about the optimal strategy and techniques for endovascular lower limb revascularization. The public, patients, and healthcare professionals have identified this area as one of high unmet need and a key priority for research in people with vascular disease^6^.

The benefits and harms of endovascular innovations are frequently debated. A recent systematic review and meta-analysis found that there was insufficient evidence to recommend different vessel preparation techniques before angioplasty, in part because of the poor reporting of RCTs^7^. It is therefore imperative that RCTs, which inform health technology adoption decisions, are optimally designed, conducted, and reported.

The authors previously published a systematic review that highlighted the poor quality of reporting of RCTs in lower limb endovascular interventions. In that study, of 112 RCTs evaluating 228 different endovascular technologies, only 21% of the endovascular interventions were reported sufficiently well to enable their replication. Standardization of the endovascular intervention was only reported in 22% of RCTs and only one study specified that adherence to the study protocol would be monitored^8,9^.

Consequently, there is an urgent need to improve the standardization and reporting of endovascular interventions for PAD in RCTs. The aim of this study was to develop a framework to assist with standardization, monitoring of adherence, and reporting of infrainguinal endovascular interventions in clinical trials to facilitate comparison between technologies and ensure the availability of robust evidence to inform optimal patient care.

## Methods

The framework was designed using international published guidance on developing robust and transparent reporting guidelines^10^. The development of the framework was underpinned by a systematic review of RCTs reporting the outcomes of endovascular interventions for infrainguinal lower limb PAD and registered on the Enhancing the QUAlity and Transparency Of health Research (EQUATOR) Network website^9,11^.

**Fig. 1:**
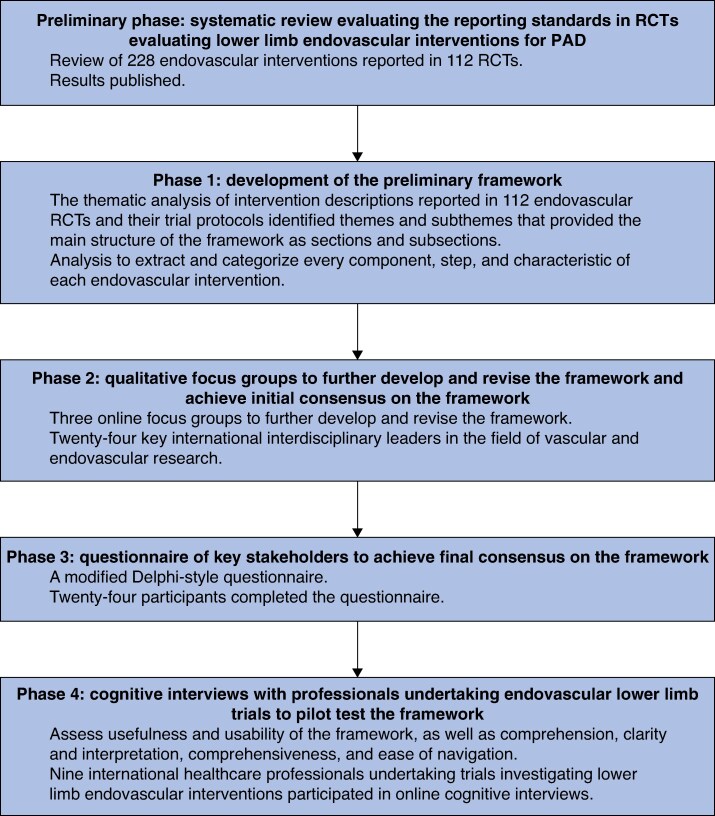
Methodology PAD, peripheral arterial disease.

There were four distinct phases of development and testing of the framework (*Fig. 1*).

### Ethical approval

The study received ethical approval from the Faculty of Health Sciences Research Ethics Committee (FREC) at the University of Bristol (reference 12228). All participants provided written consent at the start of their involvement in the study, which included recordings of interviews and focus groups and the anonymous publication of quotes.

### Phase 1: development of the preliminary framework

The aim of phase 1 was to develop an initial preliminary framework. RCTs and their protocols previously identified in the authors’ published systematic review were analysed to identify any text describing endovascular interventions^9^. After familiarization with the data, a thematic analysis was used to identify themes and subthemes. The data were systematically analysed and coded using NVivo 12 software^12^.

The qualitative content analysis was driven by a specific analytic question: identify and organize all components and steps of the endovascular intervention^13^. Consequently, the key themes identified were components of the intervention providing the main structure of the preliminary framework. This initial approach to the data analysis was purely inductive, allowing the main themes to emerge without trying to fit into a pre-existing coding frame. Once the initial key themes were identified, defining the main structure of the preliminary framework was performed using a deductive approach. The themes and subthemes identified initially at the time of the thematic analysis provided the main structure of the framework as sections and subsections.

The intervention description sections of the included papers were reanalysed and coded to extract and categorize every component, step, and characteristic of each endovascular intervention. The aim was to be as comprehensive as possible and include all of the potentially relevant details. As the data analysis progressed, new categories (or codes) were added and, if required, new categories were amended or grouped together. The interventions were generally described in chronological order. Maintaining this structure appeared to be the most logical way to describe such complex procedures composed of multiple steps and components. Once all the papers and trial protocols had been coded, these were assessed by the wider research group who reviewed and approved the final version of the preliminary framework.

### Phase 2: qualitative focus groups to further develop and revise the framework and achieve initial consensus

The aim of phase 2 was to discuss and revise the preliminary version of the framework and achieve initial consensus with key stakeholders. The preliminary framework was carefully analysed and discussed in small focus groups of key international leaders in the field of endovascular interventions for PAD. Stakeholders were identified using professional networks, journal editorial membership, and publication of trials, and they were invited to participate, representing four different groups: health professionals delivering endovascular lower limb interventions; researchers involved in (endo)vascular clinical trials; editors of journals publishing clinical trials in PAD; and medical device industry trial specialists. Discussion encouraged the refinement of the framework by adding missing elements, removing non-relevant elements, and renaming items with unclear or confusing nomenclature.

The inclusion of patient representatives in the focus groups was considered. However, given that the discussion was necessarily based on very technical aspects of endovascular interventions, extensive pre-existing knowledge was deemed essential and, consequently, patients were not included.

Three sequential focus groups were held online with an independent facilitator and, with participants’ consent, were audio recorded and transcribed.

The research team presented each section of the framework and the independent facilitator guided the discussion using a pre-prepared topic schedule (*Appendix S1*). The topic schedule was designed to encourage discussion, while investigating three main themes: completeness of components in each section of the framework; appropriateness of wording of components; and feasibility of consistent description of components.

After each meeting, a thematic analysis using constant comparative methods was conducted to analyse transcripts and meeting notes for key themes (NVivo 12 software)^14,15^. After the data analysis from each focus group, the online version of the framework was updated for discussion at the subsequent focus group.

### Phase 3: questionnaire of key stakeholders to achieve final consensus on the framework

The aim of phase 3 was to evaluate the agreement with the refined version of the framework between the participants in the focus groups and achieve a broad consensus.

A questionnaire was designed and data were collected on opinions about the agreement with each section of the framework using research electronic data capture (REDCap) tools hosted at the University of Bristol (*Appendix S2*)^16,17^. Using a standardized approach, participants were asked to rank their agreement with each section of the framework (1, completely disagree; 2, partially disagree and major changes are required; 3, partially agree and minor changes are required; 4, agree but improvements are proposed; and 5, completely agree)^18^. Additionally, each section had a free-text option to explain answers and provide suggestions for alterations or additions.

An iterative process with three rounds of questionnaires was undertaken to achieve consensus. Further rounds of questionnaires would be deemed unnecessary when an agreement level of 75% was reached^19^.

Responses to closed questions (rating scales) were summarized using descriptive statistics. Thematic analysis was used to identify patterns within the responses to free-text comments (REDCap system and NVivo 12 software).

The data from the free-text comments were systematically coded according to five predefined themes: items for inclusion in the framework; items for exclusion from the framework; items requiring rewording; items requiring clarification or definition; and items not relevant to the framework. These themes were then further categorized into: minor changes (changes that did not alter the main structure of the framework); and major changes (changes that altered the main structure of the framework).

Minor changes were assumed to be incorporated into the framework after review by the research team. Major changes, however, were expected to be re-proposed to the stakeholder group for approval in additional rounds of questionnaires.

Free text classified as ‘items requiring clarification or definition’ would then inform the completion of the practical guide to the framework.

### Phase 4: pilot testing of the framework with cognitive interviews of professionals undertaking endovascular lower limb trials

The aim of phase 4 was to evaluate the usefulness and usability of the framework and to identify potential areas for improvement or clarification.

Healthcare professionals undertaking trials evaluating lower limb endovascular technologies identified from trial registers and snowball sampling were invited to participate in a cognitive interview to pilot test the revised framework in a real-world trial setting^20,21^. The sampling was planned to continue until data saturation was reached and themes were well established, with few or no new insights gained from additional data collection. The authors estimated that about ten participants would be required. Semi-structured cognitive interviews (*Appendix S3*) were conducted online using video-conferencing software (Microsoft Teams) and were audio recorded and transcribed.

Before the interviews, participants were sent a link to the framework allowing them to review the framework ahead of the meeting. The interviewees were asked to think about a recent trial that they had been involved with and then to go through each section of the framework and consider: if they would be able to adequately describe each section in their trial; if the sections were easy to navigate; and if there was anything missing or unclear. After reviewing the whole framework in detail, additional generic questions were asked to investigate the overall utility of the framework in designing, conducting, and reporting endovascular trials.

A framework analysis of the interview transcripts was performed, given the aim to investigate predefined hypotheses regarding the usefulness and usability of the framework, and, to identify potential ‘response problems’, a deductive approach was employed^22^. Predefined codes and themes were generated based on the content related to the entire framework or specific sections and subsections.

Two predefined themes were selected to assess the usefulness and usability issues of the framework. Four additional predefined themes were selected to evaluate response problems: comprehension; clarity and interpretation; comprehensiveness; and ease of navigation.

The data from the transcripts were systematically coded according to the main themes, allowing additional subthemes to be identified during the analysis.

As the coding progressed, several subthemes were identified, including: items for inclusion in the framework; items for exclusion from the framework; items requiring rewording; items requiring clarification or definition; and items not relevant to the framework. These subthemes were then further categorized into: minor changes (changes that did not alter the main structure of the framework); and major changes (changes that altered the main structure of the framework).

Minor changes were assumed to be incorporated into the framework after review by the research team. Major changes, however, were expected to be re-proposed to the stakeholder group for approval.

Any refinements made to the analytical framework were then reapplied to the entire data set to ensure consistency and comprehensiveness in the analysis.

Any data coded as ‘items requiring clarification or definition’ would then inform the completion of the practical guide to the framework, together with data already identified in phase 3.

## Results

### Phase 1

A qualitative content analysis of the 112 RCTs (228 different endovascular interventions) identified in the systematic review identified six key themes (sections) with subthemes (subsections): expertise; setting; anaesthesia; imaging; intervention components (access; crossing lesion; treating lesion (lesion preparation, intervention, intervention optimization, bailout intervention, and treatment of non-target lesions); and closure of artery); and pharmacological interventions. The initially identified themes and subthemes provided the main structure of the framework as sections and subsections. Further analysis continued with the extraction and categorization of every component, step, and characteristic of each device or intervention into the main structure of the framework.

These were formatted into a framework and were made available in a preliminary online version on the website www.endo-star.com to allow more accessible consultation.

### Phase 2

Three sequential focus groups were conducted during the framework refinement phase. In total, 24 international key stakeholders participated in the focus groups (15 males and 9 females from Australia, Austria, Finland, France, Germany, Italy, the Netherlands, Singapore, Sweden, the UK, and the USA).

There was general agreement from the participants that there are limitations with the current reporting of endovascular trials and that a framework for guiding intervention description could be helpful. The main structure of the framework, following the chronological order of how these interventions are usually performed, was accepted by all participants as a logical and easy-to-follow approach.

It was recognized that it might be challenging to describe expertise and setting in a standardized way across different countries. However, in the expertise section, the participants recognized the importance of describing the training received, including industry-provided training and support at the time of the intervention and peer-to-peer training, the number of interventions performed in the preceding year, and specific experience in more challenging procedures, such as interventions involving below-the-knee vessels. Similarly, in the setting section, it was highlighted that a general description of the infrastructure should be sufficient and that, when describing the location of the intervention, it would be essential to specify fixed or mobile imaging, as this correlates directly with imaging quality.

The imaging section was expanded, including more recently introduced imaging modalities (such as intravascular ultrasonography) that were not used in the RCTs included in the initial analysis and allowing for future imaging methods.

Similarly, the intervention components section underwent significant modifications throughout this phase. Some of the terms used initially were considered unclear and required rewording to more recent and widely accepted terminology (for example, ‘pre-intervention treatment’ to ‘lesion preparation’). The treating lesion section was significantly expanded to include additional devices that have been introduced onto the market more recently, which, consequently, were not captured in the RCTs included in the initial analysis.

Some sections and subsections were more controversial, such as the ‘crossing lesion’ subsection and the pharmacological interventions section. The participants, in fact, recognized the practical difficulties in describing the guidewires and catheters used to cross a lesion, as a significant number could be used in an individual patient, and therefore consideration needed to be given to relevance. It was agreed that, in some specific studies, it might be relevant to clearly describe this aspect of the intervention, especially if using specific wires or catheters. In the pharmacological interventions section, the main discussion centred around which drugs should be included in the framework, considering that a multitude of different medications could affect the long-term outcomes of patients having endovascular interventions for PAD. It was agreed that concomitant medical therapy should be clearly described and reported in a trial but to only include drugs strictly relevant to the intervention in the framework.

A summary of the main points discussed in each group, any proposed changes, and points for further discussion after each focus group is presented in *Table S1*. After incorporating the final changes after the third focus group, the framework was re-proposed to the participants to achieve consensus in phase 3.

### Phase 3

The third phase aimed to achieve consensus on the framework after incorporating the final changes at the end of the focus groups and used a modified Delphi-style questionnaire completed by the initial focus group participants (*Appendix S2*). The questionnaire allowed all participants to review the refined version of the framework and provide additional comments and suggestions before the final stage.

All 24 participants completed the questionnaire in the first round, reaching universal agreement above the predefined cut-off (greater than or equal to 75%). Disagreements were only on minor issues that could be easily incorporated into the framework or were caused by misunderstanding of the framework (asking to make changes already made in the framework or asking to specify some aspects that were unclear; *Table 1*). Consequently, the decision was made that no further rounds would be required and to move towards the final phase of pilot testing.

**Table 1:** Summary of the Endo-STAR questionnaire—round 1

Section	Agreement (score 4 or 5)	Responses	Percentage
Expertise	22	24	91
Setting	24	24	100
Anaesthesia	23	24	96
Imaging	21	24	87
**Intervention components**			
Access	22	24	92
Crossing lesion	22	24	92
Treating lesion			
Lesion preparation	22	24	92
Intervention	23	24	96
Intervention optimization	22	24	92
Bailout intervention	24	24	100
Treatment of non-target lesions	24	24	100
Closure of artery	24	24	100
Pharmacological interventions	22	24	92

### Phase 4

Pilot testing of the framework was undertaken with semi-structured cognitive interviews. Ten healthcare professionals involved in clinical trials related to endovascular and vascular surgery were interviewed. Nine interviews were successfully completed (one had some technical IT issues with Microsoft Teams that could not be resolved). The international participants (8 males and 1 female) possessed extensive experience as principal investigators, associate principal investigators, investigators, or collaborators in national and international vascular and endovascular trials, including industry-sponsored and investigator-initiated trials, RCTs, and both prospective and retrospective registries. The participants provided valuable insights based on their involvement in various phases of clinical trials, ranging from writing and submitting trial protocols for approval to conducting trials, performing interventions, collecting data, and ultimately writing and submitting trial reports for publication.

#### Usefulness

The participants unanimously recognized the urgent need for standardization and improved reporting in published trials. One participant specifically highlighted the issue of poor description and standardization of comparator interventions, citing discussions about studies investigating drug-eluting *versus* plain balloons where ‘the vessel preparation was done better’ in the drug-eluting balloon interventions; they also said ‘if you used the same degree of vessel preparation to do your plain balloon angioplasty for your non-drug-eluting interventions, you would have had the same outcome’ (interviewee 2). Another participant noted that setting up an RCT can be particularly challenging and frustrating, as

‘you don't have a lot of information on how you should properly do it’ (interviewee 1). A further participant agreed that the framework would be helpful, as ‘you don't have to write everything from the beginning; you just have to adapt the study protocol based on this existing and published framework’ (interviewee 7). It was also recognized that it can be quite difficult to know how to adequately describe the intervention in a trial. One participant noted that, in examining major trials, the intervention descriptions in the methods sections were often too concise, with clear procedural steps frequently replaced by vague descriptions, such as ‘standard techniques have been used to cross the lesion’ (interviewee 2).

#### Usability issues

Some participants expressed concerns that the framework could initially seem overwhelming due to its complexity. However, they noted that the overall structure became clear and easy to follow once they became more familiar with it. They appreciated that the framework allows for flexibility, as it did not require reporting every single detail for each step and component, making it pragmatic and adaptable for the majority of trials.

#### Response problems: comprehension, clarity and interpretation, comprehensiveness, and ease of navigation

The participants agreed that the framework is relatively easy to navigate and that it is possible to adequately describe different aspects of the intervention in the setting of a clinical trial by following the framework. Some minor changes have been proposed and incorporated into the framework. Items identified as ‘requiring clarification’ have been addressed in the practical guide. A summary of the results of the cognitive interviews and the changes incorporated in the framework after focus groups are presented in *Table S2* and *Table S3*.

### Practical guide to the framework and checklist

**Fig. 2:**
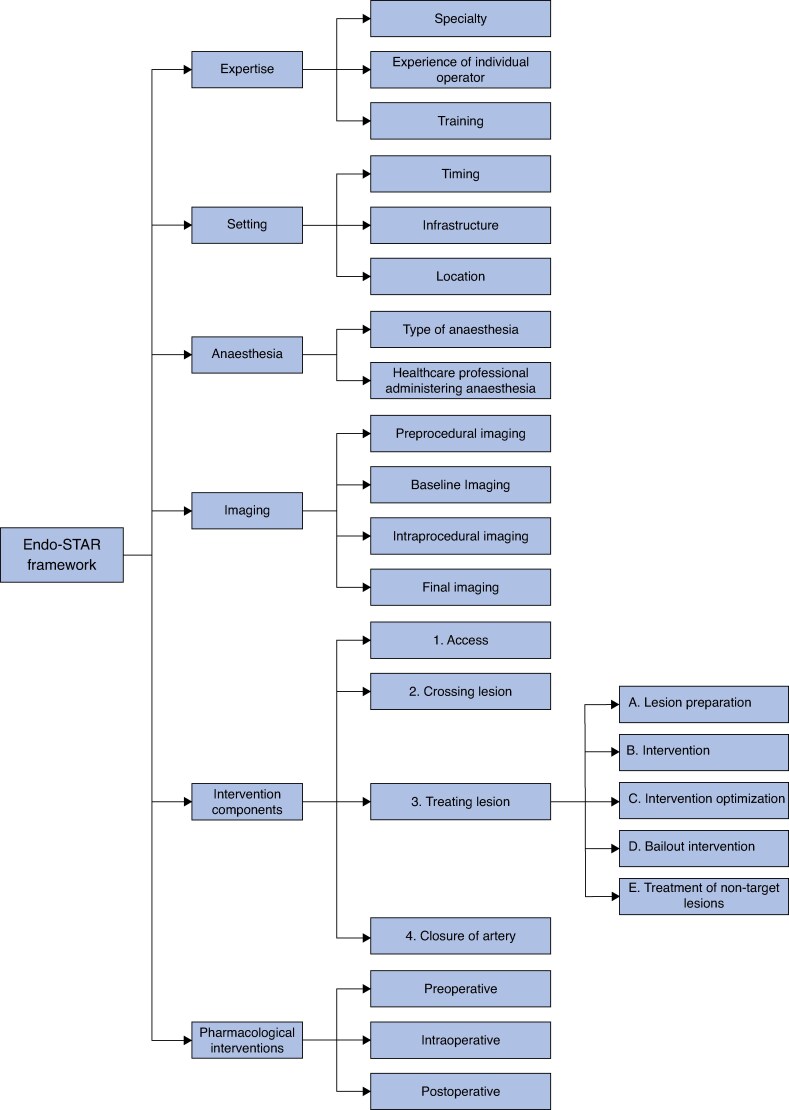
Endo-STAR framework summary

The full version of the Endo-STAR framework (*Fig. 2*) is available on the website (www.endo-star.com). The authors developed a practical guide to help healthcare professionals complete the framework and a checklist to help provide an overview of whether individual components of the endovascular intervention are described and standardized (*Appendix S4* and *Appendix S5*). The checklist also makes it possible to understand whether any trial monitors adherence to individual intervention components and how these are reported.

## Discussion

We have successfully developed and pilot tested Endo-STAR, a novel framework to optimize the design, conduct, and reporting of clinical trials of infrainguinal endovascular interventions for PAD. Endo-STAR aims to ensure that the information reported in a protocol or manuscript provides enough information for the endovascular intervention to be understood by the reader, replicated by a researcher, used by a healthcare professional to make clinical decisions for best patient care, and included in comparative effectiveness studies.

There have been concerted efforts to improve the conduct and reporting of RCTs in medicine in general and, more specifically, in studies of non-pharmacological interventions^23–25^. Whilst these have improved reporting, they have limitations in trials of some complex interventions and therefore more detailed extensions to standard reporting guidelines have been developed, for example in fertility treatments and herbal interventions, amongst others^26,27^. Whilst there have been attempts to propose a summary of reporting standards in endovascular interventions, these have not harnessed contemporary ‘gold standard’ methodologies developed by the EQUATOR Network^10^. The existing Society for Vascular Surgery (SVS) reporting standards do not consider endovascular interventions as a process involving multiple steps and components that together contribute to the final result and it does not reflect the progressive complexity of endovascular interventions involving a combination of different devices in the same procedure, making the application of this guideline difficult^28^.

The development of the Endo-STAR framework is meant to be used in parallel with the Consolidated Standards of Reporting Trials Statement for Randomized Trials (CONSORT), the Consolidated Standards of Reporting Trials Statement for Randomized Trials of Nonpharmacologic Treatments (CONSORT-NPT), and the template for intervention description and replication (TIDieR) guidelines^23–25^. Endo-STAR provides additional important details, which are essential for the comprehensive appreciation of a complex procedure, such as endovascular revascularization.

The Endo-STAR framework is an aid to design, perform, and report on high-quality clinical trials. It can be used during different stages of a clinical trial:

- Writing the trial protocol: the framework deconstructs endovascular interventions into their components and enables the standardization of each component a priori to be documented in the protocol.
- Standardizing the intervention: the framework enables standardization of an endovascular intervention that can be performed in a standardized (and replicable) way in every participant and across all trial centres.
- Monitoring of adherence to the trial protocol: enabling the research team to monitor adherence to the protocol.
- Reporting trial results: the framework will assist the research team in clearly reporting the investigated intervention when publishing trial results.

The use of the framework does not imply that every component of the endovascular intervention is recorded. However, it supports researchers to consider what components of the intervention are important when they develop the protocol (a priori) and therefore, subsequently, what should be reported. Decisions on the standardization and depth of intervention description are at the discretion of the research team based on the type of trial. For example, explanatory trials (such as ‘first in man’ or ‘pivotal device trials’) might require strict intervention descriptions and standardization, whereas pragmatic trials might prefer a more flexible approach. Key decisions in all trials will be for the research team to consider whether certain aspects of the endovascular intervention are mandatory, flexible, or prohibited.

There are limitations in any reporting guidelines. In this study, the authors suggest using established anatomical and morphological descriptions of the PAD lesions and the authors have not attempted to develop new systems, as there are already a number of these systems published, such as the Global Limb Anatomic Staging System (GLASS) and the Trans-Atlantic Inter-Society Consensus II classification (TASC II)^29–31^.

Although the authors have sought to pilot test the framework to assess its utility, the impact of the Endo-STAR framework will depend on how well it is adopted by stakeholders. If used widely and together with CONSORT, it holds the promise of improving the rigour of clinical trials and the quality of evidence underpinning the evaluation of the effectiveness of endovascular treatments of PAD. The pilot phase demonstrated the usability, but it is likely that the framework will have to evolve (as a ‘living framework’) to capture any radically innovative and disruptive endovascular technologies. Over time the framework could be harnessed to standardize the capture of data in other study designs, such as single-arm prospective studies and registries.

The full version of the Endo-STAR framework (*Fig. 2*) is available on the website (www.endo-star.com). The Endo-STAR framework should be used in conjunction with the checklist and the practical guide to the framework and completion of the checklist (*Appendix S4* and *Appendix S5*).

## Supplementary Material

znaf020_Supplementary_Data

## Data Availability

The data supporting the findings of this framework are available in the document and *Supplementary material*.
