# Correction to: Endovascular treatment of peripheral arterial disease: Endo-STAR framework for the design, conduct, and reporting of trials

**DOI:** 10.1093/bjs/znaf105

**Published:** 2025-05-07

This is a correction to: Ewa M Zywicka, Andrew J Moore, Christopher Twine, Christian-Alexander Behrendt, Michel Bosiers, Marianne Brodmann, Edward Choke, Gert J de Borst, Athanasios Diamantopoulos, Florian Enzmann, Alik Farber, Gary Ansel, Dario Gattuso, Gerard S Goh, Goueffic Yann, Shirley Jansen, Mario Landini, Anne Lejay, Michael Lichtenberg, Matthew Menard, Peter Mezes, Joseph Mills, Jane Nixon, Joakim Nordanstig, Kelly O’Connell, Baris Ozdemir, Lorenzo Patrone, Sapna Puppala, Athanasios Saratzis, Eric A Secemsky, Sigrid Nikol, Konstantinos Stavroulakis, Sabine Steiner, Martin Teraa, Isabelle Van Herzeele, Maarit Venermo, Thomas Zeller, Ronelle Mouton, Robert J Hinchliffe, Endovascular treatment of peripheral arterial disease: Endo-STAR framework for the design, conduct, and reporting of trials, *British Journal of Surgery*, Volume 112, Issue 4, April 2025, znaf020, https://doi.org/10.1093/bjs/znaf020

The eighth and thirty-first authors’ names have been corrected.

