# Supplementary material for: Endovascular treatment of peripheral arterial disease: Endo-STAR framework for the design, conduct, and reporting of trials
Source: Br J Surg. 2025 Apr 17;112(4):znaf020. doi: 10.1093/bjs/znaf020 (PMC12003854; doi:10.1093/bjs/znaf020)
Supplement: znaf020_Supplementary_Data [file znaf020_supplementary_data.docx]

**Endovascular treatment of peripheral arterial disease: The Endo-STAR framework for the design, conduct and reporting of trials**

Ewa M Zywicka^1-2^, Andrew J Moore^3^, Christopher Twine^1-2^, Christian-Alexander Behrendt ^4^, Michel Bosiers^5^, Marianne Brodmann ^6^, Edward Choke ^7^, Gert J deBorst^8^, Athanasios Diamantopoulos ^9^, Florian Enzmann^10^, Alik Farber ^11^, Gary Ansel^12^, Dario Gattuso ^13^, Gerard S Goh^14^, Goueffic Yann^15^, Shirley Jansen^16^, Mario Landini ^17^, Anne Lejay^18^, Michael Lichtenberg ^19^, Matthew Menard^20^, Peter Mezes^21^, Joseph Mills^22^, Jane Nixon^23^, Joakim Nordanstig^24^, Kelly O’Connell^25^, Baris Ozdemir^2^, Lorenzo Patrone ^26^, Sapna Puppala^27^, Athanasios Saratzis^28^, Eric A Secemsky^29^, Nikol Sigrid^30^, Konstantinos Stavroulakis^31^, Sabine Steiner^32^, Martin Teraa^33^, Isabelle Van Herzeele^34^, Maarit Venermo^35^, Thomas Zeller^36^, Ronelle Mouton^1-37^, Robert J Hinchliffe^1-2^

^1^Translational Health Sciences, Bristol Medical School, University of Bristol.

^2^Department of Vascular Surgery, North Bristol NHS Trust, Bristol, UK

^3^Musculoskeletal Research Unit, Bristol Medical School, University of Bristol, Bristol, UK

^4^Department of Vascular and Endovascular Surgery, Asklepios Clinic Wandsbek, Asklepios Medical School, Hamburg, Germany

^5^Department of Vascular Surgery, University Hospital Bern, University of Bern, Bern, Switzerland

^6^Division of Angiology, Medical University Graz, Graz, Austria

^7^Vascular and Endovascular Surgery Service, Department of General Surgery, Sengkang General Hospital, Singapore

^8^Department of Vascular Surgery G04.129, University Medical Centre Utrecht, The Netherlands

^9^Department of Interventional Radiology, Guy's and St Thomas' Hospitals, NHS Foundation Trust, London, UK

^10^Department of Vascular Surgery, Medical University of Innsbruck, Innsbruck, Austria

^11^Division of Vascular and Endovascular Surgery, Boston Medical Center, Boston University Chobanian & Avedisian School of Medicine, Boston, MA, USA

^12^Healthcare Insights, Columbus, OH, USA

^13^Fondazione Ricerca e Innovazione Cardiovascolare, Milano, Italy

^14^Department of Radiology, Alfred Hospital, Melbourne, Victoria, Australia

^15^Service de chirurgie vasculaire et endovasculaire, Groupe Hospitalier Paris St Joseph, Paris, France

^16^Curtin Medical School, Curtin University, Perth, WA, Australia; Heart and Vascular Research Institute, Harry Perkins Institute of Medical Research, University of Western Australia, Perth, WA, Australia

^17^ Clinical and Business Development Europe, Middle East & Africa, Cordis

^18^Department of Vascular Surgery, Kidney Transplantation and Innovation, Strasbourg University Hospital, Strasbourg, France

^19^Vascular Center, Klinikum Arnsberg, Arnsberg, Germany

^20^Division of Vascular and Endovascular Surgery, Brigham and Women's Hospital, Harvard Medical School, Boston, MA, USA

^21^Department of Interventional Radiology, North Bristol NHS Trust, Bristol, UK

^22^Michael E. DeBakey Department of Surgery, Baylor College of Medicine, Houston, TX

^23^Leeds Institute of Health Sciences, University of Leeds, Leeds, U.K

^24^Department of Vascular Surgery, Sahlgrenska University Hospital, Gothenburg, Sweden

^25^ Clinical Development Clinical & Medical Affairs, Philips Image Guided Therapy

^26^West London Vascular and Interventional Centre, Northwick Park Hospital, Harrow, UK

^27^ Department of Interventional Vascular Radiology, Leeds Teaching Hospitals NHS Trust, Leeds General Infirmary, Leeds, UK

^28^Department of Cardiovascular Sciences and NIHR Leicester Biomedical Research Centre, University of Leicester, Glenfield General Hospital, Leicester, UK

^29^Division of Cardiology, Beth Israel Deaconess Medical Centre, Boston, MA, USA

^30^Department of Clinical and Interventional Angiology, Asklepios Klinik St Georg, Hamburg, Germany

^31^Department of Vascular Surgery, University Hospital, LMU Munich, Munich, Germany

^32^Department of Angiology, University Hospital Leipzig, Leipzig, Germany

^33^Department of Vascular Surgery, University Medical Centre Utrecht, Utrecht, the Netherlands

^34^Department of thoracic and vascular surgery, Ghent University Hospital, Ghent, Belgium

^35^Department of Vascular Surgery, University of Helsinki and Helsinki University Hospital, Helsinki, Finland

^36^Abteilung Angiologie, Universitäts-Herzzentrum Freiburg-Bad Krozingen, Bad Krozingen, Germany

^37^Department of Anaesthesia, North Bristol NHS Trust, Bristol, UK

**Original article**

**Address for correspondence:**

Robert J. Hinchliffe

Professor of Vascular Surgery

Department of Vascular Surgery

Translational Health Sciences

Bristol Medical School

University of Bristol

2^nd^ Floor, L&R Building

Southmead Hospital

Bristol BS10 5NB

NHS: 0117 414 0841

Email: [Robert.Hinchliffe@bristol.ac.uk](mailto:Robert.Hinchliffe@bristol.ac.uk)

| Supplementary Materials- index |  |
| --- | --- |
| Appendices |  |
| Appendix 1 – Topic schedule focus groups | Pag 5 |
| Appendix 2 – Endo-STAR framework questionnaire | Pag 8 |
| Appendix 3 – Topic guide interview | Pag 16 |
| Appendix 4 – Endo-STAR Practical guide | Pag 21 |
| Appendix 5 – Endo-STAR Checklist | Pag 28 |
| Supplementary tables |  |
| Supplementary Table 1 - Changes incorporated in the EndoSTAR framework following sequential focus groups | Pag 30 |
| Supplementary Table 2 - Summary of Endo-STAR cognitive interviews | Pag 42 |
| Supplementary Table 3. Changes incorporated in the Endo-STAR framework following cognitive interviews | Pag 49 |
| References | **Pag 52** |

Standardised frameworks to improve the reporting quality of endovascular interventions for PAD in clinical trials

ENDO-STAR Framework

Focus group topic schedule

**NB This guide is necessarily provisional, as its application will depend on the experience of individual participants.**

1. Introductions and background information
   1. Welcome to the meeting from Chair
   2. Brief Introductions - attendees
   3. Explaining the aims and objectives of the study and work undertaken to date
   4. Plans for the focus group today – how it will work and any ground rules
2. ‘Expertise’ section of the framework
   - Is anything missing from this section?
     - Is it possible that this would be different for different parts of the intervention, and do we need to incorporate this?
   - Is it worded appropriately/are changes needed to the wording?
   - How practical/easy is it to describe these elements in a consistent manner? /Could it be consistently used across many studies?
3. ‘Setting’ section of the framework
   - Is anything missing from this section?
   - Is it worded appropriately/are changes needed to the wording?
   - How practical/easy is it to describe these elements in a consistent manner? /Could it be consistently used across many studies?
4. ‘Anaesthesia’ section of the framework
   - Is anything missing from this section?
   - Is it worded appropriately/are changes needed to the wording?
   - How practical/easy is it to describe these elements in a consistent manner? /Could it be consistently used across many studies?
5. ‘Imaging’ section of the framework
   - Is anything missing from this section?
   - Is it worded appropriately/are changes needed to the wording?
   - How practical/easy is it to describe these elements in a consistent manner? /Could it be consistently used across many studies?
6. ‘Intervention components’ section of the framework
   - *To go through section by section, starting with higher level headings first*
     - Access
     - Crossing the lesion
     - Treating the lesion
       - Pre-intervention treatment / lesion preparation
       - Intervention (PTA, DES, BMS, DES, AMS, AT, IVL)
         - *Any additional details to be included about tailoring of the procedure?*
     - Post-intervention treatment
     - Post-procedure imaging
     - Bailout interventions
       - - *Any additional details about bailout interventions and or indications for a bailout intervention?*
     - Treating non-target lesions
     - Closure of artery
   - Questions to ask of each section:
     - Are any components missing from this section (Access/ Crossing Lesion / Treating Lesion /Closure of Artery)?
     - Are components worded appropriately/are changes needed to the wording?
     - Are intervention components ordered appropriately?
     - How practical/easy is it to describe these components in a consistent manner? Could it be consistently used across many studies?
7. ‘Pharmacological interventions’ section of the framework
   - *To go through section by section, starting with higher level headings first.*
     - Pre-operative pharmacological interventions
     - Intra-operative pharmacological interventions
     - Post-operative pharmacological interventions
   - Questions to ask of each section:
     - Is anything missing from this section?
     - Should each medication include the following sub-components: i) agent, ii) dose, and iii) route?
     - Should each medication include alternative options if clinically appropriate, together with an indication?
     - Is it worded appropriately/are changes needed to the wording?
     - How practical/easy is it to describe these elements in a consistent manner? Could it be consistently used across many studies?
8. Overall feasibility considerations
   1. How can we prevent framework fatigue?
   2. Should we aim to incorporate this framework into CONSORT or other frameworks??
   3. How would we best ‘advertise’ the frameworks/get buy-in/ensure they are used?
   4. *Other feasibility considerations?*
9. Other thoughts or issues
10. Close of focus group
    1. Thank you
    2. Re-cap what will happen to these data and next stages

# EndoSTAR framework Consensus - Questionnaire 1

The aim of this questionnaire is to evaluate the agreement with the refined version of the framework with the participants at previously organised EndoSTAR focus groups.

As mentioned at the time of the previous meeting, the framework is meant to be comprehensive to allow addressing of all technical aspects of the investigated intervention but reporting of each detail is not compulsory and needs to be evaluated and decided by the research team.

1. **EXPERTISE**

**Please rank your level of agreement with the “Expertise” section of the framework:**

| 1 | 2 | 3 | 4 | 5 |
| --- | --- | --- | --- | --- |
| Completely disagree | Partially disagree and major changes are required | Partially agree and minor changes are required | Agree but improvements are proposed | Completely agree |

| Please explain your ranking and make any suggestions for alterations or additions (Not required if you completely agree) |
| --- |
|  |

1. **SETTING**

**Please rank your level of agreement with the “Setting” section of the framework:**

| 1 | 2 | 3 | 4 | 5 |
| --- | --- | --- | --- | --- |
| Completely disagree | Partially disagree and major changes are required | Partially agree and minor changes are required | Agree but improvements are proposed | Completely agree |

| Please explain your ranking and make any suggestions for alterations or additions (Not required if you completely agree) |
| --- |
|  |

1. ANAESTHESIA

**Please rank your level of agreement with the “Anesthesia” section of the framework:**

| 1 | 2 | 3 | 4 | 5 |
| --- | --- | --- | --- | --- |
| Completely disagree | Partially disagree and major changes are required | Partially agree and minor changes are required | Agree but improvements are proposed | Completely agree |

| Please explain your ranking and make any suggestions for alterations or additions (Not required if you completely agree) |
| --- |
|  |

1. IMAGING

**Please rank your level of agreement with the “Imaging” section of the framework:**

| 1 | 2 | 3 | 4 | 5 |
| --- | --- | --- | --- | --- |
| Completely disagree | Partially disagree and major changes are required | Partially agree and minor changes are required | Agree but improvements are proposed | Completely agree |

| Please explain your ranking and make any suggestions for alterations or additions (Not required if you completely agree) |
| --- |
|  |

1. INTERVENTION COMPONENTS

- **Access**

**Please rank your level of agreement with the “Access” section of the framework:**

| 1 | 2 | 3 | 4 | 5 |
| --- | --- | --- | --- | --- |
| Completely disagree | Partially disagree and major changes are required | Partially agree and minor changes are required | Agree but improvements are proposed | Completely agree |

| Please explain your ranking and make any suggestions for alterations or additions (Not required if you completely agree) |
| --- |
|  |

- **Crossing lesion**

**Please rank your level of agreement with the “Crossing lesion” section of the framework:**

| 1 | 2 | 3 | 4 | 5 |
| --- | --- | --- | --- | --- |
| Completely disagree | Partially disagree and major changes are required | Partially agree and minor changes are required | Agree but improvements are proposed | Completely agree |

| Please explain your ranking and make any suggestions for alterations or additions (Not required if you completely agree) |
| --- |
|  |

- **Treating lesion**
  1. Lesion preparation

**Please rank your level of agreement with the “Lesion preparation” section of the framework:**

| 1 | 2 | 3 | 4 | 5 |
| --- | --- | --- | --- | --- |
| Completely disagree | Partially disagree and major changes are required | Partially agree and minor changes are required | Agree but improvements are proposed | Completely agree |

| Please explain your ranking and make any suggestions for alterations or additions (Not required if you completely agree) |
| --- |
|  |

- 1. Intervention

**Please rank your level of agreement with the “Intervention” section of the framework:**

| 1 | 2 | 3 | 4 | 5 |
| --- | --- | --- | --- | --- |
| Completely disagree | Partially disagree and major changes are required | Partially agree and minor changes are required | Agree but improvements are proposed | Completely agree |

| Please explain your ranking and make any suggestions for alterations or additions (Not required if you completely agree) |
| --- |
|  |

- 1. Intervention optimization

**Please rank your level of agreement with the “Intervention optimization” section of the framework:**

| 1 | 2 | 3 | 4 | 5 |
| --- | --- | --- | --- | --- |
| Completely disagree | Partially disagree and major changes are required | Partially agree and minor changes are required | Agree but improvements are proposed | Completely agree |

| Please explain your ranking and make any suggestions for alterations or additions (Not required if you completely agree) |
| --- |
|  |

- 1. Bailout Intervention

**Please rank your level of agreement with the “Bailout Intervention” section of the framework:**

| 1 | 2 | 3 | 4 | 5 |
| --- | --- | --- | --- | --- |
| Completely disagree | Partially disagree and major changes are required | Partially agree and minor changes are required | Agree but improvements are proposed | Completely agree |

| Please explain your ranking and make any suggestions for alterations or additions (Not required if you completely agree) |
| --- |
|  |

- 1. Treatment of non-target lesions

**Please rank your level of agreement with the “Treatment of non-target lesions” section of the framework:**

| 1 | 2 | 3 | 4 | 5 |
| --- | --- | --- | --- | --- |
| Completely disagree | Partially disagree and major changes are required | Partially agree and minor changes are required | Agree but improvements are proposed | Completely agree |

| Please explain your ranking and make any suggestions for alterations or additions (Not required if you completely agree) |
| --- |
|  |

- **Closure of artery**

**Please rank your level of agreement with the “Closure of artery” section of the framework:**

| 1 | 2 | 3 | 4 | 5 |
| --- | --- | --- | --- | --- |
| Completely disagree | Partially disagree and major changes are required | Partially agree and minor changes are required | Agree but improvements are proposed | Completely agree |

| Please explain your ranking and make any suggestions for alterations or additions (Not required if you completely agree) |
| --- |
|  |

1. PHARMACOLOGICAL INTERVENTIONS

**Please rank your level of agreement with the “Pharmacological interventions” section of the framework:**

| 1 | 2 | 3 | 4 | 5 |
| --- | --- | --- | --- | --- |
| Completely disagree | Partially disagree and major changes are required | Partially agree and minor changes are required | Agree but improvements are proposed | Completely agree |

| Please explain your ranking and make any suggestions for alterations or additions (Not required if you completely agree) |
| --- |
|  |

ADDITIONAL COMMENTS

| Please add here any additional comments that are not covered in the sections above |
| --- |
|  |

Standardised frameworks to improve the reporting quality of endovascular interventions for PAD in clinical trials

ENDO-STAR Framework

Interview topic guide

**NB This guide is necessarily provisional, as its application will depend on the experience of individual participants.**

**Pre-interview:**

- *Brief introduction to background and rationale for study to develop a framework for lower limb endovascular interventions*
- *Brief summary of work undertaken so far to develop and update the framework (including literature review and focus groups). (Will show a very brief presentation)*
- Now, we are asking professionals who are currently designing or undertaking trials evaluating endovascular lower limb interventions to test out this framework so we can understand how they work in a real-world trial situation.
- The purpose of today’s interview is to discuss/pilot/pre-test the framework to get feedback on the content and the structure and how this would best work in a real-world trial situation.
- *Check potential participant has read and understood the information sheet and ask if they have any questions so far*
- We’ll start off by running through the consent form – I’ll switch the audio-recorder on and take verbal consent to ensure you are happy to proceed. We will then go on to the main interview.
- Are you happy for the audio-recorder to be switched on?
- *Read through statements on the consent form and ask the participant to agree/decline each statement accordingly.*
- *If consent is obtained, confirm that will now proceed with the interview. Consent form to be emailed to the participant afterwards. Remind the participant that they are free to end the interview at any time.*

**Main Interview**

**Introduction and trial context**

- Could you start off by describing your professional role?
  - Tell me about your experience working in endovascular lower limb trials.
- Could you tell me about the [NAME] trial you are in the process of designing/conducting? (*If there is more than one, ask them which they would like to think through today when going through the framework*)
  - Could you describe the trial intervention and comparator?

**Discussion on the framework**

*Briefly present the main sections of the framework. (Participant should have had a link to the framework sent in advance so can either have it up on their computer or the researcher can also share the screen*).

- Reading through the **‘Expertise’** section
  - Do the categories and items in this section make sense to you?
    - Do you understand what they mean?
    - Are there any words that are confusing?
  - Are you able to adequately describe the operator of the endovascular intervention in your trial from the categories and items included?
    - Is anything missing?
  - How clear/easy is this section to navigate?
    - What do you think about the general order of the categories and sections and how these are organized?
    - Are there parts that could be clearer?
- Reading through the **‘Setting’** section
  - Do the categories and items in this section make sense to you?
    - Do you understand what they mean?
    - Are there any words that are confusing?
  - Are you able to adequately describe the setting of the endovascular intervention in your trial from the categories and items included?
    - Is anything missing?
  - How clear/easy is this section to navigate?
    - What do you think about the general order of the categories and sections and how these are organized?
    - Are there parts that could be clearer?
- Reading through the **‘Anaesthesia’** section
  - Do the categories and items in this section make sense to you?
    - Do you understand what they mean?
    - Are there any words that are confusing?
  - Are you able to adequately describe the anaesthesia provided at the time of intervention in your trial from the categories and items included?
    - Is anything missing?
- Reading through the **‘Imaging’** section
  - Do the categories and items in this section make sense to you?
    - Do you understand what they mean?
    - Are there any words that are confusing?
  - Are you able to adequately describe the ‘imaging’ used in your trial from the categories and items included? **First thinking about the intervention and then then the comparator.**
    - Is anything missing?
  - How clear/easy is this section to navigate?
    - What do you think about the general order of the categories and sections and how these are organized?
    - Are there parts that could be clearer?

**Now moving onto the Intervention Components section which is the most detailed**

- Reading through the **‘Access’** section
  - Do the categories and items in this section make sense to you?
    - Do you understand what they mean?
    - Are there any words that are confusing?
  - Are you able to fully describe the “access” for endovascular interventions in your trial from the categories and items included? **First thinking about the intervention and then then the comparator.**
    - Is anything missing?
  - How clear/easy is this section to navigate?
    - What do you think about the general order of the categories and sections and how these are organized?
    - Are there parts that could be clearer?
- Reading through the **‘Crossing lesion’** section
  - Do the categories and items in this section make sense to you?
    - Do you understand what they mean?
    - Are there any words that are confusing?
  - Are you able to fully describe the “crossing lesion” step for endovascular interventions in your trial from the categories and items included? **First thinking about the intervention and then then the comparator.**
    - Is anything missing?
  - How clear/easy is this section to navigate?
    - What do you think about the general order of the categories and sections and how these are organized?
    - Are there parts that could be clearer?
- Reading through the **‘Treating lesion’** section
  - Do the categories and items in this section make sense to you?
    - Do you understand what they mean?
    - Are there any words that are confusing?
  - Are you able to fully describe the “treating lesion” component for endovascular interventions in your trial from the categories and items included? **First thinking about the intervention and then then the comparator.**
    - Is anything missing?
  - How clear/easy is this section to navigate?
    - What do you think about the general order of the categories and sections and how these are organized?
    - Are there parts that could be clearer?
- Reading through the **‘Closure of artery’** section
  - Do the categories and items in this section make sense to you?
    - Do you understand what they mean?
    - Are there any words that are confusing?
  - Are you able to fully describe the “closure of artery” for endovascular interventions in your trial from the categories and items included? **First thinking about the intervention and then then the comparator.**
    - Is anything missing?
  - How clear/easy is this section to navigate?
    - What do you think about the general order of the categories and sections and how these are organized?
    - Are there parts that could be clearer?
- Reading through the **‘Pharmacological interventions’** section
  - Do the categories and items in this section make sense to you?
    - Do you understand what they mean?
    - Are there any words that are confusing?
  - Are you able to fully describe the “pharmacological interventions” in your trial from the categories and items included? **First thinking about the intervention and then then the comparator.**
    - Is anything missing?
  - How clear/easy is this section to navigate?
    - What do you think about the general order of the categories and sections and how these are organized?
    - Are there parts that could be clearer?

**Intervention standardization and monitoring**

Thank you for thinking through all of that. Another area that we would like to build into this framework is how they could help trialists decide when and how individual components of the interventions should be standardized and monitored within a trial.

So first, they would help the triallists to fully describe all the different components of their interventions within the trial protocol (everything we have just been through), and then from this detailed description, triallists would decide which of these components need to be standardized and monitored within their particular trial.

We haven’t currently included any sections or items related to standardization or monitoring within the framework.

- Would you find it helpful for these elements to be included?
- How might we embed these elements within the framework in a user-friendly way?

**Overall including Format/layout**

- Is there anything else that is missing from the framework that you haven’t already mentioned?
- How do you find the overall format/layout of the framework?
- What would be the best format/layout for accessing the framework?
- Any suggestions for how it could be designed differently?

**Overall utility**

- Would this framework be helpful to you in designing, conducting and reporting an endovascular lower limb trial?
- How likely would you be to use it at:
  - Design stage? (E.g Helping you to think through all the important elements and which should be standardized?
  - During trial conduct (monitoring)
  - During trial reporting?
- How could it be more helpful?

**Wrap-up**

- Thank you for your time
- *Ask for any known colleagues/names that might be suitable to interview*

| 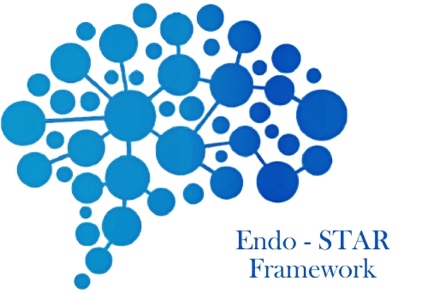 | | | | The Endo-STAR Framework* a practical guide to the framework and completion of the checklist |
| --- | --- | --- | --- | --- |
| The Endo-STAR framework is a practical aid to help design, perform and report clinical trials of lower limb endovascular interventions for peripheral arterial disease. It can be used at different stages of the clinical trial and for different purposes:   - Writing the trial protocol - Standardising the intervention - Monitoring adherence to the trial protocol - Reporting trial results   The Endo-STAR framework checklist is available in appendix 4, and the full version of the framework is available on the Endo-STAR framework website (www.endo-star.com). This document is a guide on how to use the framework in conjunction with the checklist. | | | | |
| General principles on how to use the framework and checklist:  -For the purpose of the intervention description, it is assumed that the intervention begins when the patient arrives in the operating room and ends when the patient leaves the operating room.  -It is not compulsory to describe, standardise, monitor and report all steps and components of the intervention. However, it is important for the research team to consider all of these individual components of the endovascular intervention “a priori” from the design stage of the trial and document the decision clearly in the trial protocol. Please note that the same considerations should be applicable to both the main intervention and the comparator intervention.  -The Endo-STAR framework Checklist should be used as a tool to check that these points have been considered by the research team and document the outcomes. The checklist can be downloaded as appendix 4 or from the Endo-STAR framework website and can be included as part of the protocol publication or an online appendix.  In an explanatory trial (such as a first in vivo trial), the research team might be willing to describe, standardize and report each step and component of the procedure.  In a pragmatic trial the research team might instead be keen to leave some components of the intervention (for example the type of access or the type of closure device) at the discretion of the operator while standardizing and monitoring other steps and components to ensure that the investigated procedure is performed in a similar way between all participants across all trial centres and can be replicated by others.  - If a trial is already ongoing similar consideration should be applicable at the reporting stage where the research team should consider reporting an as accurate as possible description of the investigated intervention.  - If the information is too long and complex to be described in the primary paper, alternative formats should be used (separate publication of trial protocol, appendix), and details of where this can be obtained should be provided in the primary paper. | | | | |
| Section | | | |  |
| 1. Expertise | | | | **This section provides guidance on how to describe the expertise, training and support for the operator. The term “operator” refers to the person who delivers the intervention.**  It is important to describe the expertise, including the specialist's background or clinical specialty (vascular surgeon, interventional radiologist, cardiologist, angiologist), pre-existing specific skills, experience, details of any training specific to the intervention and procedural competence / support. More than one operator is acceptable. However, defining the primary and secondary operator while describing their expertise is required.  This can be used to describe the eligibility criteria for care providers at the design stage and to describe those who provided the intervention at the reporting stage. |
| 1. Setting | | | | **This section provides guidance on how to describe the setting in which the intervention is being investigated.**  The term “setting” describes the circumstances in which the intervention is performed. This can be variable based on the timing of the procedure (elective / urgent / emergency), the infrastructure (different types of hospitals or ambulatory centres) and, more specifically, the specific environment or location (operating room, hybrid theatre, interventional radiology / cardiology / angiology suite) and equipment (fixed or mobile imaging).  This can be helpful to describe the eligibility criteria for centres at the design stage and to describe where the interventions were performed at the reporting stage. |
| 1. Anaesthesia | | | | **This section provides guidance on how to describe the anaesthesia being used while performing the intervention.**  More than one type of anaesthesia is possible, as well as a combination of different modalities (for example, local anaesthesia and sedation or local anaesthesia and a popliteal nerve block in patients with severe rest pain). |
| 1. Imaging | | | |  |
|  | | Pre-procedural imaging | | The initial subsection “Pre-procedural imaging” refers to the imaging performed before the intervention to plan and decide revascularization options, characterize and classify the lesions. These includes, for example, preoperative CTA, MRA, and arterial Duplex scans.  It should also be considered to establish a priori and clearly document based on which imaging modality or standard, the reference vessel diameter and/or length will be measured to select the most appropriate device. This is often done either based on pre-procedural imaging or baseline imaging. |
|  | | Baseline imaging | | The subsection “Baseline imaging” refers to the initial imaging performed at the beginning of the procedure to assess the anatomy and the lesions requiring intervention before performing any treatment.  The most common imaging modality is a diagnostic angiogram, but other modalities can also be utilized, such as external and intravascular US or Optical Coherence Tomography.  It should be considered performing the baseline imaging in a standardised mode, considering the appropriate modality for the visualization and evaluation of all relevant anatomical segments and including details such as specific views for angiography (antero-posterior and lateral for example) or the type of contrast being used. |
|  | | Intra-procedural imaging | | The subsection “Intra-procedural imaging” refers to imaging performed between different steps or devices, to guide them and evaluate their results.  In addition to the details provided about the modality, it is important to consider specifying eventual adjuncts such as rulers or road map, especially if those may demonstrate technical advantages in the whole procedure. |
|  | | Final imaging | | The subsection “Final imaging” refers to the imaging performed at the end of the intervention to assess the final result.  This is often represented by a final completion angiogram with complete views of the distal arterial tree, but it is important to clearly specify which modality has been used for this assessment with all the relevant details as defined in the framework. |
| 1. Intervention components | | | |  |
|  | 1. Access | | | This subsection provides guidance on how to describe access for the intervention. It is suggested to specify the type of access (percutaneous/surgical), the location or more than one location if applicable (anatomical location and if ipsilateral/contralateral or both), whether it is antegrade or retrograde, if the access is image guided ( US guided or xray guided) or not, and finally providing details about the sheath being used or specifying if it has not been used.  *In pragmatic trials, for example, the research team might decide to leave this step of the intervention at the operator's discretion and not specify or standardise it. In other trials, it might be of interest to evaluate complications related to the point of arterial access, and in this case a careful description of this step might be crucial*. |
|  | 1. Crossing lesion | | | This subsection provides guidance on how to describe how a lesion is being crossed at the time of intervention. It is important to specify if there is a pre-defined crossing modality such as intraluminal, subintimal, mixed, antegrade or retrograde, and specific crossing techniques.  It is suggested to provide further details about the types of wires and catheters, and to specifically provide details about the use of any specific wires (CTO wire) catheters or devices. However, it is recognised that the description and recording of each wire and catheter used for the intervention may not be feasible and realistic. |
|  | 1. Treating lesion | | |  |
|  |  | | 1. Lesion Preparation | The “lesion preparation” subsection provides guidance on how to describe the steps taken to prepare the lesion for the main intervention.  It is proposed to specify whether the lesion preparation is mandatory for all participants, recommended or allowed at the operator's discretion, or required in only specific situations (for example, to allow the passage of devices).  The modality of lesion preparation, such as balloon pre-dilatation, atherectomy, intravascular lithotripsy, thrombectomy, or other interventions, should be specified, and further details for each modality should be clarified as proposed in the framework. If the research team does not allow any lesion preparation techniques or devices, this should be clearly specified in the trial protocol.  It is suggested to specify how the results of the lesion preparation have been assessed before proceeding with the main intervention.  *For example, if in a trial all patients should have a specific lesion preparation decided by the research team, this should be clearly described in the trial protocol, standardised, monitored and reported.* |
|  |  | | 1. Intervention | The “intervention” subsection provides guidance on how to describe the main intervention performed to treat the lesion.  Devices currently available on the market are already included, but it is accepted that newer devices will need to be included in the future.  It is acceptable to use one or more devices, as a combination of multiple devices can be required while evaluating complex procedures. It is then suggested to clarify in which order the devices were used, provide specific details for each device as specified in the framework, and clarify if the results were evaluated between each device. |
|  |  | | 1. Intervention optimisation | The “intervention optimisation” subsection provides guidance on how to describe additional interventions that might be required to improve the result of the intervention.  It is suggested to specify which intervention could be required to optimise the intervention, if it is mandatory, recommended or allowed and specify how the results of this step will be evaluated.  *For example, a non-fully deployed stent might require a post-dilatation by the trial protocol, while in another trial, the post-dilatation might be considered mandatory for each intervention.* |
|  |  | | 1. Bailout Intervention | The “Bailout Intervention” subsection provides guidance on how to describe what interventions are acceptable when it is necessary to deal with eventual complications.  It is suggested to explain the indications for a bailout intervention, such as flow-limiting dissection, perforation, residual stenosis, vessel spasm, thrombosis or distal embolization.  It is proposed to describe which interventions would be acceptable (prolonged balloon inflation, stenting, thrombectomy, thrombolytic or vasodilating agents or other interventions) and which intervention would not be acceptable, and how the results would be re-evaluated.  *For example, in a trial evaluating a drug eluting balloon, stenting might be considered an appropriate bailout intervention, but drug eluting stents might not be allowed by the trial protocol.* |
|  |  | | 1. Treatment of non-target lesions | The “Treatment of non-target lesions” subsection provides guidance on how to describe concomitant treatment of additional non-target lesions if allowed by the trial protocol.  These lesions can be inflow, outflow or other vessels on the same side or the contralateral limb.  It is proposed to specify which interventions are permitted and which are not.  *For example, in a trial evaluating drug-eluting balloons, it might be acceptable to treat another lesion with a plain balloon, but it might not be allowed to use another drug-eluting device.* |
|  | 1. Closure of artery | | | The “Closure of artery” section provides guidance on how to describe the final step of the intervention. This can be done with direct external pressure, a closure device or a surgical closure.  *It is possible for a research team not to describe and standardise this step, leaving this to the discretion of the operator, while in another trial, it might be appropriate to describe the device being used if, for example, evaluating access complications.* |
| 1. Pharmacological interventions | | | | The “Pharmacological Interventions” section provides guidance on how to describe the pharmacological treatment related to the procedure administered before, at the time and after the procedure.  This section does not exhaustively list all drugs the participants might be taking in the perioperative period, as this should still be captured while reporting baseline demographic data. |

| 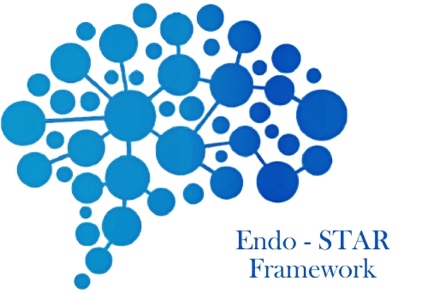 | | | The Endo-STAR Framework* checklist | | | |
| --- | --- | --- | --- | --- | --- | --- |
| Section | | | Description  (Yes/No) | Standardisation  (Yes/No) | Adherence Monitoring  (Yes/No) | Reporting  (Yes/No) |
| 1. Expertise | | |  |  |  |  |
| 1. Setting | | |  |  |  |  |
| 1. Anaesthesia | | |  |  |  |  |
| 1. Imaging | | |  |  |  |  |
| 1. Intervention components | | |  |  |  |  |
|  | 1. Access | |  |  |  |  |
|  | 1. Crossing lesion | |  |  |  |  |
|  | 1. Treating lesion | |  |  |  |  |
|  |  | 1. Lesion Preparation |  |  |  |  |
|  |  | 1. Intervention |  |  |  |  |
|  |  | 1. Intervention optimisation |  |  |  |  |
|  |  | 1. Bailout Intervention |  |  |  |  |
|  |  | 1. Treatment of non-target lesions |  |  |  |  |
|  | 1. Closure of artery | |  |  |  |  |
| 1. Pharmacological interventions | | |  |  |  |  |

***We strongly recommend using the checklist in conjunction with The Endo-STAR framework (**[**www.endo-star.com**](http://www.endo-star.com)**) and the Endo-STAR framework practical guide.**

| Description | Check if the description of each section of the framework is adequate for the stage of the clinical trial:   - Adequate description in the trial protocol to ensure it is extremely clear what intervention is being investigated. - Adequate description for the practitioners to perform the intervention as intended by the research team. - Adequate description to allow monitoring of adherence to the trial protocol to ensure the intervention is performed by different practitioners in different trial centres as intended by the research team. - Adequate description of the intervention to interpret the results, compare them with results of other studies, and replicate the intervention in clinical practice. |
| --- | --- |
| Standardisation | Check that the standardisation of each section of the framework has been considered “a priori” and that the decision to standardise or not each section or subsection of the framework has been documented in the trial protocol.    Ensure that the “standard” to adhere to is clearly defined by the research team. |
| Adherence Monitoring | Check that the monitoring of the adherence to the trial protocol over the course of the trial has been considered “a priori” and documented in the trial protocol, performed over the course the trial, and reported at the reporting stage. |
| Reporting | Check that at the time of reporting trial results, the investigated intervention is clearly described and details about standardisation and adherence monitoring are also provided. |

| **Supplementary Table 1 . Changes incorporated in the framework following sequential focus groups** | | | | | | | | |
| --- | --- | --- | --- | --- | --- | --- | --- | --- |
| **First Focus group** | | | | | | | | |
|  | | | | | | Discussions | Changes | Outstanding points |
| Expertise | | | | | | - Training grade- “difficult to have a description/classification that will fit every country” - Expertise – “will need expertise in that specific field (Expertise in lower limb PAD). Still no good answer if it would be useful to specify years of experience or numbers of procedures for example” - Specific training in using a new device or performing a new intervention - Industry-provided training and support in the room at the time of intervention |  | *How practical/easy is it to describe “the experience” in a consistent manner across different countries?* |
| Setting | | | | | | - Clarify the difference between “infrastructure and location” - Infrastructure – “difficult to have a description/classification that will fit every country” - Considering adding all different types of locations such as theatre, hybrid suite, cath lab, IR suite or make a distinction based on the type of imaging ( fixed or mobile) as this is correlated to a different image quality? - Add timing of intervention (elective/ urgent/emergency) | - Clarified that “infrastructure” is the type of hospital/environment where the procedure is performed while “location” is the actual room/theatre/suite where the procedure is performed. - Timing of intervention (elective/ urgent/emergency) |  |
| Anaesthesia | | | | | | - “Who administered the anaesthesia? Anaesthetic support or directly administered by the Cardiologist/Radiologist” | - Operator administering the anaesthesia |  |
| Imaging | | | | | | - Imaging classification “Pre -procedure – Post procedure is confusing? Add intra-procedural imaging? Better wording – Final imaging rather than Post procedure” | - IVUS for sizing - Visual estimate for sizing under angiography - CO2 as contrast media |  |
| Intervention components | Access | | | | |  | - US guided access - Radial artery access - Multiple access locations - Sheath-less approach for retrograde puncture |  |
|  | Crossing lesion | | | | | - Difficulties in classifying the type of crossing of the lesion: “Sometimes not clear if intraluminal or subintimal or subadventitial“ - Difficulties in specifying all sort of wires and catheters in an intervention: “Dedicated CTO wires for example or workhorse wires or standard wires and specific devices?” | - Added unknown/unspecified crossing | *Is wire size a relevant component in this section of the framework?* |
|  | Treating lesion | | | Lesion preparation | |  | - Atherectomy - Specialty balloons like scoring balloons - Shockwave lithotripsy | *Should we include in the framework at this stage emerging new techniques such as laser atherectomy or new lithotripsy devices?* |
|  |  |  |  | Intervention | | - Difficult in documenting “How much of the lesion is treated? Spot/partial/complete” - Discussions if including wire size for each device or not | - What type of atherectomy device (make/type of device) - Laser atherectomy - New Lithotripsy devices - Distal protection device also used without atherectomy | *Should we specify in the framework to include the make and model of each device, some devices or leave it at the discretion of the research team?*  *Should we specify in this framework if the device used was licensed for the procedure or used off license ( for example devices borrowed from cardiology for peripheral interventions)?* |
|  |  |  |  | Intervention optimisation | |  | - Remove pos-intervention treatment |  |
|  |  |  |  | Bailout intervention | |  | - Thrombus Aspiration - Spasm – with vasodilators or treatment |  |
|  |  |  |  | Treatment of non-target lesions | |  |  |  |
|  | Closure of artery | | | | |  | - Surgical closure |  |
| Pharmacological Interventions | | | | | | - Discussions about the drugs to be included as part of intervention and drugs that should be reported as having long term impact on patients but not strictly relevant to the procedure itself. | - Prolonged Thrombolysis - Prolonged IV Heparin infusion | - Addition of statins, diabetic meds, Ace inhibitors or only anticoagulation/antiplatelets |
| **Second Focus group** | | | | | | | |  |
|  | | | | | | Discussions | Changes | Outstanding points |
| Expertise | | | | | | - Expertise of individual operator not institution | - More than one operator to be included - Volume of intervention in last 12 months - Peer to peer training - Predefine number of procedures performed before practice in trial – to account for learning curve - Completion of training rather than grade ( before training, one to 5 years, 5 to 10, over 10 years) |  |
| Setting | | | | | |  | - Remove Cath Lab/Hybrid - Keep fixed imaging versus mobile imaging as quality is different |  |
| Anaesthesia | | | | | | Discussion if this section is needed. Generally strong feeling that anaesthesia may impact outcome.  Difference between operator or anaesthetist but no need to differentiate between different type of anaesthetists. Considered including expertise of anaesthetist but not decided that not very relevant. |  |  |
| Imaging | | | | | |  | - Add BTK above the ankle, BTK below the ankle and pedal views (BTK -ATA and BTK BTA) - Method rather than modality - Include both Iodinated and CO2 contract and combination of two. - Include external ultrasound rather than duplex - EVUS - Change wording to pre-interventional imaging or baseline imaging. |  |
| Intervention components | | Access | | | |  |  |  |
|  | | Crossing lesion | | | | Discussion about reporting of all wires and catheters or only “the most escalated wire” | - Diameter of wire (Calibre) and coating of wire (weight of wire) and CTO or not | *How best to describe the wire used for crossing the lesion? Do we need more details about wires and catheters? Do we need all wires on only the last/most escalated wire used for the procedure?* |
|  | |  | | | Lesion preparation |  | - Change name from pre-intervention treatment to lesion preparation - Include Speciality balloon with specification of type (Focal force balloon – including cutting balloon, scoring balloon, high pressure non-compliant balloon, chocolate balloon - Include mechanical thrombectomy - Atherectomy : Include mechanism and divide into categories and size of the catheter (Rotational/Orbital/ Directional/Laser - Change inflation time from standard to “per protocol” | *Do we need to include any other category of speciality balloons for lesion preparation?*  *Do we need to include more details regarding Atherectomy devices?* |
|  | |  | | | Intervention | - Remove wire size from devices as usually given by instructions for use. - Atherectomy and Lithotripsy - suggestion to keep in only as vessel preparation but not everyone in agreement - Make and model of device - definitely for drug eluting devices and stents. Same for atherectomy and lithotripsy | - DBC – Needs further information (because of dose-dependent issues): number of balloons used for each individual (Length, Diameter, nflation time, Inflation pressure, Brand) - DES – Needs further information. number of stents implanted, length, diameter, Brand. | *Are there any sections that currently requires excessive level of detail? Anything that might be appropriate to remove or simplify?* |
|  | |  | | | Intervention optimisation | - Discussion about the difference between just suboptimal stent deployment (optimisation) and complication (clarified this part includes formal complications) | - Added again the Post-intervention treatment section but renamed to Intervention optimisation - Rename Thrombus aspiration to mechanical thrombectomy |  |
|  | |  | | | Bailout intervention |  |  |  |
|  | |  | | | Treatment of non-target lesions | Considered Including results and freedom from complication, but capturing results or complications is outside of the scope of the framework. |  |  |
|  | | Closure of artery | | | |  |  |  |
| Pharmacological Interventions | | | | | | Ongoing discussion about which drugs should be included in the framework | Remove analgesia | *Is it appropriate to record only anticoagulants and antiplatelets as postoperative pharmacological interventions? Or should we also record statins, diabetic and hypertensive medications with the risk of increasing the complexity of the framework?* |
| **Third Focus group** | | | | | | | |  |
|  | | | | | | Discussions | Changes |  |
| Expertise | | | | | |  | - Clarify which type of training – Specialist training. - Technically successful procedures rather than successful - Specific experience in BTK/SFA |  |
| Setting | | | | | |  | - Urgent category – between elective and emergency. |  |
| Anaesthesia | | | | | | - Discussion if anaesthesia should be included in the framework. |  |  |
| Imaging | | | | | |  | - AP/Lateral views for angiography (at least 30 degree) |  |
| Intervention components | Access | | | | |  | - Brachial access/ or Other - Bi-directional access |  |
|  | Crossing lesion | | | | | - Ongoing discussion about specifying the type of wires as might not be relevant to know the type of wire while is important to know the modality of crossing. Agreement that can be captured if the specific research group wishes to. |  |  |
|  |  | | Lesion preparation | | |  | - Remove chocolate balloon- to not encourage use of a very specific device. - Lithotripsy – too much details (As per IFU) |  |
|  |  | | Intervention | | | - Discussion if include details such as Polymer based or polymer-free DES, Eccipient or not. Agreement that this information would be captured under brand and name of device. | - Include covered stents - Difference between bare metal stents (laser cut nitinol stents) and interwoven nitinol stents and covered stents. |  |
|  |  | | Intervention optimisation | | |  |  |  |
|  |  | | Bailout intervention | | |  | - Change type of stents and add covered stents. - Indication – Other/Impaired flow(too generic) |  |
|  |  | | Treatment of non-target lesions | | |  |  |  |
|  | Closure of artery | | | | |  | - Time of manual compression - Type name/brand closure device |  |
| Pharmacological Interventions | | | | | | Discussion about drugs to be included, agreement that there is no need for a too granular approach specifying all possible drugs as not relevant as part of intervention description. Other drugs should be reported in the demographic section or while specifying eventual compliance with best medical therapy as per international guidelines. |  |  |

| **Supplementary Table 2. Summary of Endo-STAR cognitive interviews** | | | | |
| --- | --- | --- | --- | --- |
| **Themes** | **Questions** | **Section** | **Agreement** | **Comments** |
| Comprehension  Clarity and Interpretation Comprehensiveness | Are you able to adequately describe this section in your trial? | Expertise | Yes (9/9) | “nicely captures what is the operators experience” (Interviewee 1)  “If there's a way to add multiple members to the team, I think that'd be helpful”(Interviewee 2)  “all these information are very important. Including the training, I think it's a key point and this never reported.” (Interviewee 8) |
|  |  | Setting | Yes (9/9) | “we have different some of the different elements for the infrastructure in the US little bit different, but you captured a lot of our unique areas … like the outpatient setting which is unique to us.” (Interviewee 9) |
|  |  | Anaesthesia | Yes (9/9) |  |
|  |  | Imaging | Yes (8/9) | “You cannot standardise a trial if you don't standardise the information that you received during the baseline angiogram or baseline imaging.” (Interviewee 7) |
|  |  | Intervention Components |  | “that's good. that's that looks really good.” (Interviewee 10) |
|  |  | Access | Yes (9/9) |  |
|  |  | Crossing lesion | Yes (9/9) |  |
|  |  | Treating lesion | Yes (9/9) |  |
|  |  | Closure | Yes (9/9) |  |
|  |  | Pharmacological interventions | Yes (9/9) |  |
| Ease of Navigation | Is this section easy to navigate? | Expertise | Yes (9/9) |  |
|  |  | Setting | Yes (9/9) |  |
|  |  | Anaesthesia | Yes (9/9) |  |
|  |  | Imaging | Yes (8/9) |  |
|  |  | Intervention Components |  |  |
|  |  | Access | Yes (9/9) |  |
|  |  | Crossing lesion | Yes (9/9) |  |
|  |  | Treating lesion | Yes (9/9) |  |
|  |  | Closure | Yes (9/9) |  |
|  |  | Pharmacological interventions | Yes (9/9) |  |
| Comprehensiveness | Is anything/g missing? |  | Changes included in Supplementary Table 3 |  |
| Comprehension | Is anything unclear requiring clarification? |  | Changes included in Supplementary Table 3 | “All made sense to me, so this was I think this is this is excellent.” (Interviewee 10) |
| Usefulness | Do you think that that this framework will be helpful in designing, conducting or reporting and endovascular trials? |  |  | “I think that we need more consistency in terms of how we do this and everybody creates a new framework set that this is an important objective way of collecting these data.” (Interviewee 3)  “For the future to have nice results and outcomes, it would be good to have certain aspects of the framework to be mandatory, and other aspects to be optional.” (Interviewee 5)  “it's really a great framework and there are so many things that that I'm sure that a lot of these people that did big trials have not thought about. So yeah, I think this will be extremely helpful in planning trials in setting up protocols and also reporting” (Interviewee 2)  “anyone putting a trial together that hasn't done it before will find it very helpful” (Interviewee 1)  “if you have a framework which where you can just refer to it's much easier and faster to create a complete protocol from beginning on and not always going back to say oh I forgot this I forgot that so it's a helpful tool for conducting and setup the protocol” ((Interviewee 2)  “it's helpful because you don't have to write everything from beginning, so you just have to adapt the study protocol based on this existing and published framework” (Interviewee 7) |
| Usability issues | Is this the general structure of the framework clear? |  | Yes (9/9) | “I think the framework look to overall be representative about how we think about the elements of a trial. I think that it was nicely organized and compartmentalized.” (Interviewee 10)  “I think that this framework would work.” (Interviewee 3)  “it will be helpful in reporting and designing as well, but you may have to make some data mandatory and some may be non-mandatory to keep it feasible for the researchers to complete.” (Interviewee 5) |
| Usability issues | Is this layout relatively straightforward/easy to be used? |  |  | “it seemed like it was a clean, organized framework.” (Interviewee 6 )  “This looks nice. It's easy to use.”  “it's very detailed and I think that's going to be the main problem always with the surgeons, isn't it that if they're going to complete it, but I think it's a great guide helping people to set up a study.” (Interviewee 8)  “It's the first glimpse is kind of overwhelming because you have all these points at the sub points and the sub points and at the beginning you kind of feel overwhelmed by the by the sheer amount of things you want to look into.” (Interviewee 2) |
| Usability issues | Any suggestions about how to make it easier for people to use it? |  |  | “ Implement the framework into case report forms (CRF)” (Interviewee 1)  “Make it available online” (Interviewee 5)  “Support from national and international societies” (Interviewee 7)  “Drop down menu with name of wires to make it easier?” ((Interviewee 4)  “make sure that they have to type as less as possible” (Interviewee 10) |

| **Supplementary Table 3. Changes incorporated in the Endo-STAR framework following cognitive interviews** | | | | | | |
| --- | --- | --- | --- | --- | --- | --- |
| Expertise | | | Add more than one operator |  |  |  |
| Setting | | | Subdivide fixed imaging into interventional suite and hybrid theatre |  |  |  |
| Anaesthesia | | |  |  |  |  |
| Imaging | | | Move sizing standard before baseline imaging and call it Pre -procedural imaging | Rename to baseline classification and lesion characterisation (lesion length, location, calcifications, CTO, Presence of thrombotic material, TASC/GLASS) | Add Road Map fluoroscopy to adjuncts |  |
| Intervention components | Access | |  |  |  |  |
|  | Crossing lesion | | Specify wire tip weight | Specific techniques used like: CART, reverse CART, rendezvous technique with the use of balloon ( specific techniques but without using a specific device) | Specify difference between selective catheter and supportive catheter |  |
|  | Treating lesion | Lesion preparation | Change mechanical thrombectomy to endovascular thrombectomy and add the various devices: pharmaco-mechanical thrombectomy, aspiration thrombectomy, rotational thrombectomy, etc and specify brand and model |  |  |  |
|  |  | Intervention | Add cycles/impulses to lithotripsy | Atherectomy - where do we start and do we finish - add length treated | Add Other atherectomy modalities | Add specialty balloons to treatment not only vessel prep as are often used as standalone treatment |
|  |  | Intervention optimisation |  |  |  |  |
|  |  | Bailout intervention | Add Bail out - surgical conversion | Add bleeding ( access bleeding) in bailout | Change mechanical thrombectomy to endovascular thrombectomy and add the various devices: pharmaco-mechanical thrombectomy, aspiration thrombectomy, rotational thrombectomy, etc and specify brand and model |  |
|  |  | Treatment of non-target lesions |  |  |  |  |
|  | Closure of artery | |  |  |  |  |
| Pharmacological Interventions | | |  |  |  |  |

**References**

1. GBD 2019 Peripheral Artery Disease Collaborators. Global burden of peripheral artery disease and its risk factors, 1990-2019: a systematic analysis for the Global Burden of Disease Study 2019. Lancet Glob Health. 2023 Oct;11(10):e1553-e1565.

1. Eid MA, Mehta K, Barnes JA, Wanken Z, Columbo JA, Stone DH, Goodney P, Mayo Smith M. The global burden of peripheral artery disease. J Vasc Surg. 2023 Apr;77(4):1119-1126.e1
2. Song, P.; Rudan, D.; Zhu, Y.; Fowkes, F.J.I.; Rahimi, K.; Fowkes, F.G.R.; Rudan, I. Global, Regional, and National Prevalence and Risk Factors for Peripheral Artery Disease in 2015: An Updated Systematic Review and Analysis. Lancet. Glob. Heal. 2019, 7, e1020–e1030, doi:10.1016/S2214-109X(19)30255-4.
3. Saeedi, P.; Petersohn, I.; Salpea, P.; Malanda, B.; Karuranga, S.; Unwin, N.; Colagiuri, S.; Guariguata, L.; Motala, A.A.; Ogurtsova, K.; et al. Global and Regional Diabetes Prevalence Estimates for 2019 and Projections for 2030 and 2045: Results from the International Diabetes Federation Diabetes Atlas, 9th Edition. Diabetes Res. Clin. Pract. 2019, 157, 107843, doi:10.1016/j.diabres.2019.107843.
4. Goodney PP, Beck AW, Nagle J, Welch HG, Zwolak RM. National trends in lower extremity bypass surgery, endovascular interventions, and major amputations. J Vasc Surg. 2009 Jul;50(1):54-60
5. <https://www.jla.nihr.ac.uk/priority-setting-partnerships/vascular-conditions/vascular-conditions-top-10s.htm>
6. Nugteren MJ, Welling RHA, Bakker OJ, Ünlü Ç, Hazenberg CEVB. Vessel Preparation in Infrapopliteal Arterial Disease: A Systematic Review and Meta-Analysis. J Endovasc Ther. 2024 Apr;31(2):191-202
7. Zywicka EM, Elliott L, Twine CP, Mouton R, Hinchliffe RJ. Protocol for a systematic review of reporting standards of lower limb endovascular interventions in peripheral arterial disease. Syst Rev. 2023 Feb 15;12(1):20.
8. Zywicka EM, McNally E, Elliott L, Twine CP, Mouton R, Hinchliffe RJ. Exploring the Reporting Standards of Randomised Controlled Trials Involving Endovascular Interventions for Peripheral Arterial Disease: A Systematic Review. Eur J Vasc Endovasc Surg. 2024 Jan;67(1):155-164.
9. <https://www.equator-network.org/toolkits/developing-a-reporting-guideline/>
10. EQUATOR Network. <https://www.equator-network.org/library/reporting-guidelines-under-development/reporting-guidelines-under-development-for-clinical-trials/#ENDOSTAR>
11. Lumivero (2017) NVivo (Version 12) [www.lumivero.com](http://www.lumivero.com)
12. Elo S, Kyngäs H. The qualitative content analysis process. J Adv Nurs. 2008 Apr;62(1):107-15.
13. Caelli K, Ray L, Mill J. ‘Clear as mud’: toward greater clarity in generic qualitative research. International journal of qualitative methods. 2003;2(2):1-13.
14. Cooper S, Endacott R. Generic qualitative research: a design for qualitative research in emergency care? Emergency Medicine Journal. 2007;24(12):816.
15. Harris PA, Taylor R, Thielke R, Payne J, Gonzalez N, Conde JG. Research electronic data capture (REDCap)--a metadata-driven methodology and workflow process for providing translational research informatics support. J Biomed Inform. 2009 Apr;42(2):377-81
16. Harris PA, Taylor R, Minor BL, Elliott V, Fernandez M, O'Neal L, McLeod L, Delacqua G, Delacqua F, Kirby J, Duda SN; REDCap Consortium. The REDCap consortium: Building an international community of software platform partners. J Biomed Inform. 2019 Jul;95:103208.
17. Moore AJ, Wylde V, Whitehouse MR, Beswick AD, Walsh NE, Jameson C, Blom AW. Development of evidence-based guidelines for the treatment and management of periprosthetic hip infection. Bone Jt Open. 2023 Apr 1;4(4):226-233
18. Morisset J, Johannson KA, Jones KD, Wolters PJ, Collard HR, Walsh SLF, Ley B; HP Delphi Collaborators. Identification of Diagnostic Criteria for Chronic Hypersensitivity Pneumonitis: An International Modified Delphi Survey. Am J Respir Crit Care Med. 2018 Apr 15;197(8):1036-1044.
19. Robinson, O. C. (2013). Sampling in Interview-Based Qualitative Research: A Theoretical and Practical Guide. Qualitative Research in Psychology, 11(1), 25–41. https://doi.org/10.1080/14780887.2013.801543
20. Wolcott MD, Lobczowski NG. Using cognitive interviews and think-aloud protocols to understand thought processes. Curr Pharm Teach Learn. 2021 Feb;13(2):181-188.
21. Gale NK, Heath G, Cameron E, Rashid S, Redwood S. Using the framework method for the analysis of qualitative data in multi-disciplinary health research. BMC Med Res Methodol. 2013 Sep 18;13:117.
22. Moher D, Hopewell S, Schulz KF, Montori V, Gøtzsche PC, Devereaux PJ, Elbourne D, Egger M, Altman DG; Consolidated Standards of Reporting Trials Group. CONSORT 2010 Explanation and Elaboration: Updated guidelines for reporting parallel group randomised trials. J Clin Epidemiol. 2010 Aug;63(8):e1-37.
23. Boutron I, Moher D, Altman DG, Schulz KF, Ravaud P; CONSORT Group. Extending the CONSORT statement to randomized trials of nonpharmacologic treatment: explanation and elaboration. Ann Intern Med. 2008 Feb 19;148(4):295-309.
24. Hoffmann TC, Glasziou PP, Boutron I, Milne R, Perera R, Moher D, Altman DG, Barbour V, Macdonald H, Johnston M, Lamb SE, Dixon-Woods M, McCulloch P, Wyatt JC, Chan AW, Michie S. Better reporting of interventions: template for intervention description and replication (TIDieR) checklist and guide. BMJ. 2014 Mar 7;348:g1687.
25. Gagnier JJ, Boon H, Rochon P, Moher D, Barnes J, Bombardier C; CONSORT Group. Reporting randomized, controlled trials of herbal interventions: an elaborated CONSORT statement. Ann Intern Med. 2006 Mar 7;144(5):364-7
26. Harbin Consensus Conference Workshop Group; Conference Chairs; Legro RS, Wu X; Scientific Committee; Barnhart KT, Farquhar C, Fauser BC, Mol B. Improving the reporting of clinical trials of infertility treatments (IMPRINT): modifying the CONSORT statement†‡. Hum Reprod. 2014 Oct 10;29(10):2075-82.
27. Stoner MC, Calligaro KD, Chaer RA, Dietzek AM, Farber A, Guzman RJ, et al. Reporting standards of the Society for Vascular Surgery for endovascular treatment of chronic lower extremity peripheral artery disease: Executive summary. J Vasc Surg 2016;64:227e8.
28. Conte MS, Bradbury AW, Kolh P, White JV, Dick F, Fitridge R, Mills JL, Ricco JB, Suresh KR, Murad MH; GVG Writing Group. Global vascular guidelines on the management of chronic limb-threatening ischemia. J Vasc Surg. 2019 Jun;69(6S):3S-125S
29. Norgren L, Hiatt WR, Dormandy JA, Nehler MR, Harris KA, Fowkes FG; TASC II Working Group; Bell K, Caporusso J, Durand-Zaleski I, Komori K, Lammer J, Liapis C, Novo S, Razavi M, Robbs J, Schaper N, Shigematsu H, Sapoval M, White C, White J, Clement D, Creager M, Jaff M, Mohler E 3rd, Rutherford RB, Sheehan P, Sillesen H, Rosenfield K. Inter-Society Consensus for the Management of Peripheral Arterial Disease (TASC II). Eur J Vasc Endovasc Surg. 2007;33 Suppl 1:S1-75.
30. Jaff MR, White CJ, Hiatt WR, Fowkes GR, Dormandy J, Razavi M, Reekers J, Norgren L. An update on methods for revascularization and expansion of the TASC lesion classification to include below-the-knee arteries: A supplement to the inter-society consensus for the management of peripheral arterial disease (TASC II): The TASC steering committee. Catheter Cardiovasc Interv. 2015 Oct;86(4):611-25.
